# Trapped fourth ventricle: a rare complication in children after supratentorial CSF shunting

**DOI:** 10.1007/s00381-020-04656-w

**Published:** 2020-05-07

**Authors:** Ahmed El Damaty, Ahmed Eltanahy, Andreas Unterberg, Heidi Baechli

**Affiliations:** 1 Department of Neurosurgery, Heidelberg University Hospital, Im Neuenheimer Feld 400, 69120 Heidelberg, Germany; 2 School of Medicine, Mansoura University, Mansoura, Egypt; 3 Department of Experimental Medical Sciences, Faculty of Medicine, Lund University, Lund, Sweden

**Keywords:** Cerebrospinal fluid, Hydrocephalus, Overdrainage, Post-hemorrhagic hydrocephalus, Prematurity, Trapped fourth ventricle

## Abstract

**Purpose:**

Trapped fourth ventricle (TFV) is a well-identified problem in hydrocephalic children. Patients with post-hemorrhagic hydrocephalus (PHH) are mostly affected. We tried to find out predisposing factors and describe clinical findings to early diagnose TFV and manage it.

**Methods:**

We reviewed our database from 1991 to 2018 and included all patients with TFV who required surgery. We analyzed prematurity, cause of hydrocephalus, type of valve implanted, revision surgeries, modality of treatment of TFV, and their clinical examination and MRI imaging.

**Results:**

We found 21 patients. Most of patients suffered from PHH (16/21), tumor (2/21), post-meningitis hydrocephalus (2/21), and congenital hydrocephalus (1/21). Seventeen patients were preterm. Seven patients suffered from a chronic overdrainage with slit ventricles in MRI. Thirteen patients showed symptoms denoting brain stem dysfunction; in 3 patients, TFV was asymptomatic and in 5 patients, we did not have available information regarding presenting symptoms due to missing documentation. An extra fourth ventricular catheter was the treatment of choice in 18/21 patients. One patient was treated by cranio-cervical decompression. Endoscopic aqueductoplasty with stenting was done in last 2 cases.

**Conclusion:**

Diagnosis of clinically symptomatic TFV and its treatment is a challenge in our practice of pediatric neurosurgery. PHH and prematurity are risk factors for the development of such complication. Both fourth ventricular shunting and endoscopic aqueductoplasty with stenting are effective in managing TFV. Microsurgical fourth ventriculostomy is not recommended due to its high failure rate. Early detection and intervention may help in avoiding fatal complication and improving the neurological function.

## Introduction

A trapped fourth ventricle (TFV) is a well-identified problem in children with hydrocephalus. The fourth ventricle can become isolated due to multiple causes, most frequently after hemorrhage, infection, or congenital anomalies [1]. Although a specific pathophysiological explanation remains unclear, post-hemorrhagic hydrocephalus (PHH) may result from an inflammatory response (e.g., ependymitis, arachnoiditis) with attendant occlusion of the cerebral aqueduct of Sylvius and foramina of Luschka and Magendie or scarring and obstruction of the surface absorptive mechanisms [2, 3]. Isolation of the fourth ventricle likely occurs from obstruction of the cerebral aqueduct of Sylvius and the fourth ventricle outflow tracts. Usually, it presents delayed after the first shunt procedure. This period is very variable and ranges in the literature from 4 weeks to 12 years [4, 5]. Delayed diagnosis of TFV can lead to severe neurological dysfunction and/or death [3, 6, 7].

The available treatment modalities are variable; for example, open surgery (fourth ventriculocisternostomy) to cerebrospinal fluid (CSF) diversion (transcerebellar, transaqueductal, transforaminal, or transcortical) and endoscopic procedures (aqueductoplasty ± stenting whether anchored or non-anchored, cystoventricular stenting) [4, 8–12]. Conservative management without surgery has been shown to be effective for patients with unequivocal clinical and radiographic stability [3, 7]. The literature remains limited regarding the frequency, natural and surgical histories, and long-term clinical and radiographic outcomes of TFV [1, 3, 6, 7].

Understanding the CSF physiology is still evolving and incomplete. In the traditional bulk flow model described by Dandy over a century ago, CSF is secreted by the choroid plexus epithelium in the cerebral ventricles, flows into the subarachnoid spaces, and enters the cerebral venous system via the arachnoid granulations [13, 14]. Oi and Di Rocco [15] proposed a new classification for hydrocephalus with a special reference to the CSF circulation in the minor CSF pathway, i.e., “minor pathway hydrocephalus,” differentiating the conventional classification by Dandy (communicating and non-communicating) or Russell (non-obstructive and obstructive) as “major pathway hydrocephalus.” Minor pathway hydrocephalus is disruption of CSF flow and reabsorption in newly elucidated channels in the brain parenchyma, which involves deep vascular structures and lymphatic channels. Iliff et al. [16] further characterized this pathway in rodents using in vivo two-photon imaging and coined the term “glymphatic system.” There were many previous trials to elucidate the role of the lymphatic system in CSF circulation [17].

## Materials and methods

We reviewed our database retrospectively starting from 1991 to 2018 after the approval of our ethical committee for carrying on the desired study. Consents from patients were not required due to the retrospective nature of the study. We included all patients under 18 years of age (pediatric population) by which trapped fourth ventricle was diagnosed, irrelevant of the etiology, and was managed surgically. We reviewed the gender, birth status whether preterm or full-term, primary hydrocephalus cause, type of shunt implanted by the first operation, valve pressure gradient, and clinical and radiological follow-up before and after surgery.

All patients were managed multidisciplinary, including neonatal intensive care and/or from neurological pediatrician. All data regarding history, neurological examination, postoperative course (from the initial CSF shunting procedures), including time to event which represents time between VP shunt insertion and development of TFV requiring treatment, were reviewed and analyzed.

All patients underwent preoperative CT and/or MR imaging using standard T1- and T2-weighted images. The studies were independently reviewed by a neuroradiologist and pediatric neurosurgeon to determine the degree of aqueduct and fourth ventricular outlet patency as well as the presence of fourth ventricular enlargement and brainstem compression. Surgery was indicated in patients with clinical symptoms suggesting brain stem dysfunction, and/or progressive enlargement of the fourth ventricle. In cases of clear radiological evidence of TFV with brain stem compression, the parents were advised for surgery even if the patient was asymptomatic or were severe disabled children where it is difficult to assess the neurological function. The surgical options we considered were insertion of a separate fourth ventricular catheter transcerebellar and to connect it to the already implanted VP shunt before the implanted valve through a Y-connector, endoscopic aqueductoplasty with stenting in cases of TFV with absence of slit ventricles and a short-segment obstruction of the aqueduct, or a microsurgical fourth ventriculocisternostomy. Method of surgical treatment was chosen according to the surgeon’s preference and MRI findings. The procedure was considered successful if at follow-up there was either improvement in clinical symptoms, improvement in fourth ventricle size, reduction in the preoperative brain stem distortion, or arrest of the progression of the fourth ventricle dilation.

We implemented statistical tests using SAS version 9.1 (SAS Institute Inc., Cary, NC) through Fisher’s exact test to analyze the contingency table including PHH and prematurity and calculate the *p* value for each correlation to find whether it is significant or not. A *p* value of 0.05 or less was considered to be significant.

## Results

### Patients characteristics

We reviewed our neurosurgical database for all cases of TFV managed surgically and found totally 21 patients (including the 11 patients born in our center) fulfilling the inclusion criteria, 10 males and 11 females. Age at time of surgery varied from 2 months to 15 years with a mean of 5.1 years. Time to event which represents the time between the insertion of VP shunt and development of TFV requiring treatment varied from 2 months to 15 years, with a mean of 4.3 years. The follow-up ranged from 24 up to 324 months, with a mean of 162 months.

**Fig. 1:**
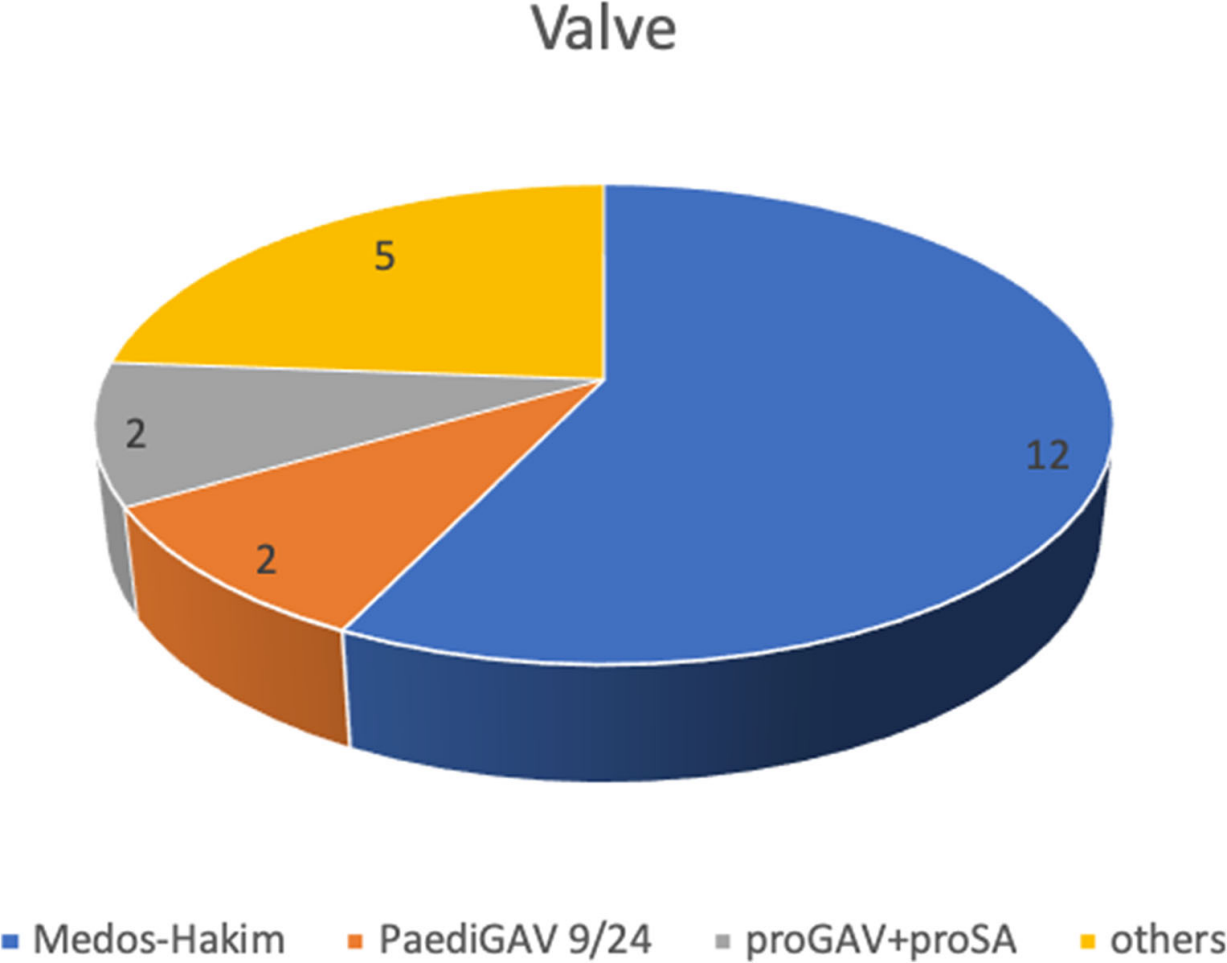
Distribution of valve used

According to etiology, most of our patients suffered from post-hemorrhagic hydrocephalus (16/21), tumor-related hydrocephalus (2/21), post-meningitis hydrocephalus (2/21), and congenital hydrocephalus (1/21) (for details see Table 1). Regarding prematurity, 17 out of 21 were delivered preterm, *p* value = 0.008. Medos-Hakim programmable valve was the most implanted valve (12/21) (see Fig. 1). Thirteen patients out of 21 were symptomatic with radiological evidence of TFV. Symptoms were mostly unspecific but denote brain stem dysfunction; headache with projectile vomiting in 5/21, somnolence in 5/21, tonic seizures in 3/21, ataxia in 2/21, nystagmus in 2/21, bulbar palsy in 2/21, abducens paresis in 1/21, intermittent bradycardia in 1/21, progressive spasticity in 1/21, hypertensive crisis in 1/21; in 3 patients, TFV was asymptomatic but with clear radiological evidence of entrapment of the fourth ventricle and massive brainstem compression; in 5 patients, we did not have available information regarding presenting symptoms due missing documentation after management of TFV in a foreign country (see Table 2). These 5 patients were operated abroad as they were not living in our country and then immigrated to our country and continued their follow-up visits in our institute until now. We noticed in our long-term cohort a decline in the occurrence of TFV over the last years after the introduction of the anti-siphon device in VP shunt systems due to the marked decrease in overdrainage.

**Table 1:** Etiology of hydrocephalus in patients

Etiology of hydrocephalus	Number
PHH	16 (76%)
Tumor-related	2 (9.5%)
Post-meningitis	2 (9.5%)
Congenital	1 (4.7%)
Total	21

**Table 2:** Patients’ symptoms

Symptoms	Number (percentage %)
Headache with projectile vomiting	5 (23.8%)
Somnolence	5 (23.8%)
Tonic seizures	3 (14.3%)
Ataxia	2 (9.5%)
Nystagmus	2 (9.5%)
Bulbar symptoms	2 (9.5%)
Abducens paresis	1 (4.7%)
Intermittent bradycardia	1 (4.7%)
Progressive spasticity	1 (4.7%)
Hypertensive crisis	1 (4.7%)
No symptoms	3 (14.7%)
N/A	5 (23.8%)

### Surgical treatment

**Fig. 2:**
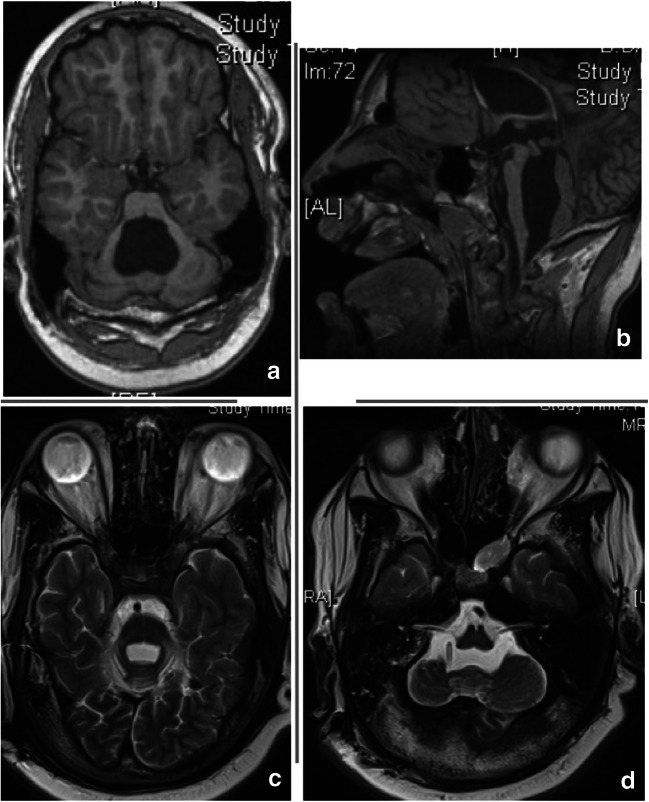
MR images demonstrating a TFV in a preterm with PHH, came at age of 15 years with fatigue and somnolence. **a**, **b** Axial and sagittal MR T1-weighted images showing ballooning of the fourth ventricle with brainstem compression. **c**, **d** Axial T2-weighted images 5 years after insertion of the fourth ventricular catheter and connection to implanted VP shunt

**Fig. 3:**
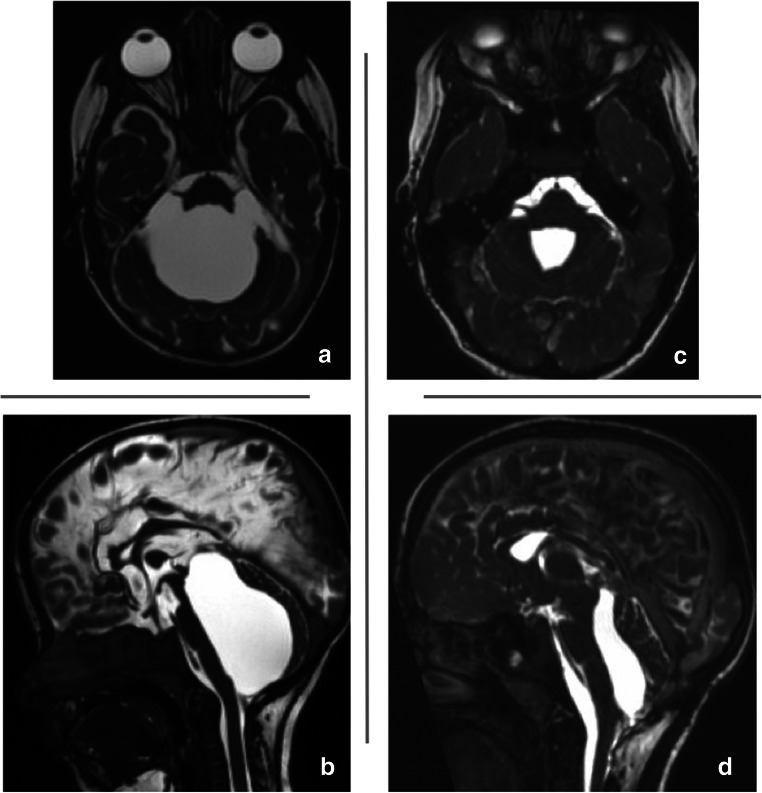
MR images demonstrating a TFV in a preterm with PHH, came at age of 5 years for a regular follow-up. **a**, **b** Axial and sagittal MR T2-weighted images showing ballooning of fourth ventricle with massive brainstem compression. **c**, **d** Axial T2-weighted images 5 years after failed cranio-cervical decompression and insertion of fourth ventricular catheter and connection to implanted VP shunt

**Fig. 4:**
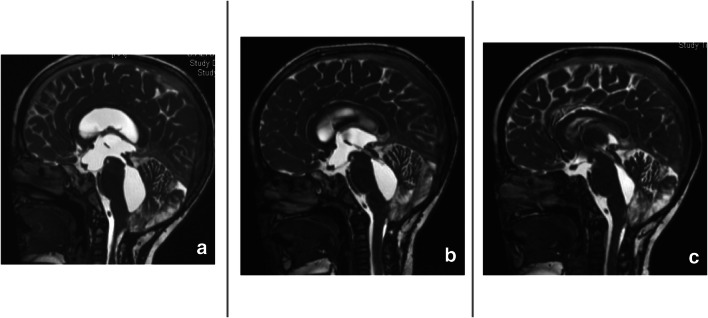
MR images demonstrating TFV in a child after resection of medulloblastoma. Patient presented with fatigue, bilateral abducens paresis, and ataxia denoting VP shunt dysfunction as well as TFV. For the purpose of the planned intrathecal chemotherapy, aqueductoplasty with stenting was done to ensure application of the chemotherapy to the local tumor bed in the fourth ventricle. **a** Mid-sagittal CISS MR image showing the TFV. **b**, **c** Mid-sagittal CISS MR images showing the stent in place immediate postoperative and 1.5 years later respectively

All patients showed improvement of their presenting symptoms, we have missing data in 5 patients whom their operations were done abroad due to missing documentation, and those patients came after surgery for follow-up in our center without recurrent symptoms of TFV. Surgery in the form of a separate fourth ventricular catheter was the treatment of choice in 18 out of 21 patients (Fig. 2). One patient was treated by cranio-cervical decompression (fourth ventriculocisternostomy) but unfortunately did not show any improvement of his presenting symptoms; he was operated 2 weeks later, and a fourth ventricular catheter connected to the implanted VP shunt system was inserted (Fig. 3). An endoscopic aqueductoplasty with stenting was considered and performed in the last two cases using a single ventricular catheter placed anterograde from the lateral ventricle (Fig. 4). No direct complications related to the surgery of the TFV were reported except for the failed improvement after cranio-cervical decompression in one patient. Three patients needed replacement of the fourth ventricular catheter after 4, 14, 8 years respectively due to its slippage outside the fourth ventricle with the normal head growth and decrease in size of TFV. These patients developed radiologically enlarging TFV again. One patient needed a fourth ventricular catheter revision 1 year after insertion due to proximal occlusion. We had noticed in one patient that after the TFV decompression and reduction of its size that the catheter lied in the fourth ventricle but the tip touching the brainstem, but the patient did not suffer from any related symptoms, so we decided to manage it conservatively without surgical correction. We considered only the complications related to TFV surgery as some of our patients were re-operated during follow-up due to valve dysfunction or distal catheter occlusion but not related to the TFV. One patient died after 10 years of treatment of his TFV due to an acute shunt dysfunction in a rehabilitation facility.

### Slit ventricles

Seven patients out of 21 suffered from a chronic overdrainage during follow-up with a radiological evidence of slit ventricles. The preoperative CT or MR images in all patients showed a clear evidence of entrapment of the fourth ventricle with brain stem compression determined by both the neuroradiologist and the pediatric neurosurgeon and proved through severe obliteration to complete absence of the prepontine cistern as a sign of brainstem ventral displacement. All postoperative images showed decrease of the size of the TFV and release of the brain stem compression.

## Discussion

In our study, we found that the symptoms of TFV are very diverse and could be easily missed from clinicians. PHH and prematurity represent risk factors for developing TFV. PHH causes inflammation of the ependyma which possibly leads to scarring of the microvasculature and altering the CSF absorption mechanisms; also through scarring, mechanical obstruction of the inlet and outlet foramina of the fourth ventricle may occur which leads to isolation of the fourth ventricle. Preterm infants are prone to development of germinal matrix hemorrhage which may develop into PHH later on; also their skull morphometry is prone to develop a symptomatic TFV due to their small posterior fossa. TFV may also occur following surgery of tumors of the fourth ventricle due to possible scarring after surgery with subsequent isolation of the fourth ventricle.

### Limitations of the study

In our cohort, we described the incidence of TFV requiring surgical intervention in preterm infants with VP shunt due to PHH and it was 29.7%. We could not mention the number of patients diagnosed with TFV who were managed conservatively which is considered a limitation of our study as we included only the patients who were treated surgically. Pomeraniec et al. [3] described similar results in a smaller cohort of 8 consecutive cases involving pediatric patients with TFV following VP shunting for IVH due to prematurity between 2003 and 2012. The frequency of TFV following VP shunting for neonatal PHH was found to be 15.4%. Three (37.5%) patients presented with symptoms of posterior fossa compression and were treated surgically. Of the 5 patients treated conservatively, 80% experienced stable ventricular size, and 1 patient experienced a slight increase (3 mm) on imaging. All of the nonsurgical patients showed stable to improved clinical examinations over the follow-up period.

Another pertinent concern raised by this study is the question whether it is better to observe these children with large TFV or drain them by either a shunt or stent. Currently, there are no evidence-based answers to this question; there can be valid arguments made for and against the intervention. In a distinct subgroup of patients, i.e., infants and children with cerebral palsy and delayed development, a progressively dilating TFV can cause significant brainstem distortion without causing recognizable symptoms [12]. The symptoms of feeding difficulties, apathy, somnolence, and seizures often can be ascribed to associated causes, thus missing a symptomatic TFV. The presence of persistent or progressive brain stem compression with or without distortion with an aqueduct inlet obstruction is concerning, for it potentially can result in gradual worsening of symptoms which may go undetected. Similarly, the dilation of the fourth ventricle with signs of compression of the brainstem indicates a significantly raised intra-fourth ventricular pressure, whereas the clinician is uncertain about its contribution to overall symptoms in a delayed child. Draining the TFV would potentially reduce such concern. However, placing a stent or shunt also has surgical risks, and those risks have to be carefully weighed before deciding the procedure. In our opinion, we believe that radiological evidence of progressive enlargement and persistent brain stem compression with or without symptoms is an indication for decompression of the TFV.

### Relation between PHH and TFV

Klebe et al. [18] tried to explain the development of PHH following germinal matrix hemorrhage (GMH) with or without intraventricular hemorrhage (IVH). PHH after GMH may be obstructive in nature during the acute phase due to the hematoma, but generally develops as chronic communicating hydrocephalus into adolescence and adulthood. Many GMH/IVH studies suggest that PHH is a consequence of obstructions within the ventricular system and subarachnoid drainage pathways due to thrombi, gliosis, and fibrosis. These are not merely obstructing CSF passages but also are altering barrier dynamics in the microvasculature and ependymal lining which result in development of PHH. These findings go with our results, as most of our patients (76%) suffered from PHH, the effect of blood products on the ependymal lining or the subarachnoid space causes progressive inflammation and secondary scarring of the surrounding structures which may lead to entrapment of the fourth ventricle [18]. Functional collapse of the walls of the aqueduct occurs due to change in the pressure gradient across the tentorium, induced by the lateral ventricular shunt creating an upward displacement of the aqueduct combined with a lowered intraventricular pulse pressure and brain compliance in the shunted ventricle. Accordingly, CSF flow through the aqueduct is impaired and occlusion may occur [19].

### Relation between chronic overdrainage and TFV

Chronic CSF overdrainage modifies the dynamics and structure of the cerebral venous system leading to pathological cerebral venous overdrainage through loss of the “Starling resistor” which accordingly affects the coupling between compartments and hence the regulation of intracranial pressure [20]. CSF overdrainage from cerebral ventricles can produce their passive collapse as a sequel of the “negative” ICP [21]. This post-shunting ventricular collapse may cause a cerebellar “sag” leading to transient occlusion of posterior fossa bridging veins and secondary venous infarction with consequent adhesions, which may participate in the pathology of TFV [20]. In our cohort, radiological evidence of chronic overdrainage was found in only 33% (7/21) of our patients. But we also noticed a decline in occurrence of TFV over the last years after introduction of the anti-siphon device. When we look back on the incidence of TFV in our cohort and its correlation to using anti-siphon device, we noticed that during the first one-third (1991–2000), we encountered 6 patients; during the second period (2001–2010), we encountered 9 patients; and after using anti-siphon device as standard in our practice the number of TFVs dropped back to 6 patients during the third period (2011–2018). Despite these results, the role of chronic overdrainage in the development of TFV remains questionable as it was only proven radiologically in one-third of the patients.

### Relation between skull morphometry and TFV

During infancy, the growing skull and the developing brain interact with each other and eventually achieve the adult shape and size of the head and brain. A preterm infant was demonstrated to have an occipito-frontal and inferior-superior elongation and a temporal narrowing of the skull together with a flattening of the occipital skull base [22]. This may predispose to developing a small posterior fossa and hence predisposing to symptomatic TFV. In our cohort, most of our patients (17/21) representing 81% were preterm, which in our opinion, according to the previous explanations, could be a risk factor for developing symptomatic TFV.

### Symptomatology of TFV

TFV is most commonly seen in children with PHH with occluded outlet foramina resulting in enlargement of all ventricles. A well-functioning lateral VP shunt causes the lateral and third ventricles to be well-decompressed. TFV can be associated with overt clinical signs or be asymptomatic and detected at routine follow-up neuroimaging [12]. The common clinical features include truncal instability, poor feeding, dysconjugate eye movements, or somnolence. It is common that these patients suffer from developmental delay which makes the diagnosis depending on symptoms more difficult. The indications for decompression of a TFV are controversial. Most authors recommend surgery only for symptomatic patients [4, 23–25]. In older children and adults, it is often easier to assess if the dilated fourth ventricle is symptomatic. However, in infants or younger children with developmental delay, this is often difficult, and the condition can be easily missed until a later stage. In our cohort, we considered surgery for TFV when the fourth ventricle remained persistently dilated with significant brain stem compression and when a progressive dilatation was demonstrated during the follow-up even in the absence of symptoms. In our study, we noticed that most of the older children were symptomatic for the condition, whereas the asymptomatic group was predominantly infants, younger children, or patients with severe disability i.e., cerebral palsy.

### Complications related to surgery

Complications related to the insertion of ventricular catheter in the fourth ventricle have been reported. These include infection, mechanical irritation of the brainstem, malfunction, and overdrainage. Cranial nerve palsy is a rare complication and has been mostly described in children [26, 27]. In our patients, we intended to place the ventricular catheter in the fourth ventricle relatively short to avoid brainstem injury as it usually occurs after decompression of the TFV and the relief of the pressure that the brainstem is displaced dorsally and comes near to the newly placed catheter which sometimes causes brainstem injury with possible cranial nerve deficits. We did not encounter that complication in our cohort. On the contrary, due to our adopted technique, we needed to replace the catheter in 3 patients due to its slippage outside the TFV.

### Shunt versus stent

Mohanty and Manwaring [12] tried to assess the efficacy of aqueductoplasty with stenting in managing TFV and compare the outcome with fourth ventricular shunting. They reported on 25 patients (children and adults) who were surgically treated for TFV. Out of 25 patients, 12 were symptomatic, while 13 were asymptomatic. Nineteen underwent aqueductoplasty + stenting, whereas in 6 patients, fourth ventricular shunt was performed. Patients with an identified short-segment aqueductal stenosis were considered for stent placement; those with long-segment aqueductal obstruction underwent fourth ventricular shunting. They concluded that both fourth ventricular shunting and endoscopic stent placement are effective in managing TFV. The extent of aqueductal obstruction and degree of ventriculomegaly are often the deciding factors in choosing the management option. This goes with our results; we had symptomatic TFV in 13 out of 21 patients. Aqueductoplasty with stenting was done in the last 2 patients with short-segment aqueductal obstruction.

To our knowledge, our study represents the largest series of patients in a pediatric age group with trapped fourth ventricle from a single institution with long-term clinical and radiographic outcomes who were managed surgically following neonatal post-hemorrhagic hydrocephalus and other etiologies. We tried to clarify, in our opinion, the pathophysiology of such finding and how to manage. Besides the well-known high incidence of TFV in patients suffering from PHH and managed with VP shunt, we describe that such complication may occur following surgery of tumors of the fourth ventricle due to possible scarring of the foramina Luschka and Magendie. Most of these patients receive VP shunt during the course of disease which leads, in similar way to PHH shunting, to functional collapse of the walls of the aqueduct and subsequent aqueductal stenosis. Further research is highly required to investigate the details of the CSF circulation especially with the enormous development in the field of neuroimaging, i.e., dynamic MRI. Accordingly, future management of neonatal hydrocephalus may change markedly.

## Conclusion

Diagnosis of clinically symptomatic TFV and its treatment is a challenge in our practice of pediatric neurosurgery due to the diversity of presenting symptoms. We found that post-hemorrhagic hydrocephalus which is usually occurring with prematurity is a risk factor for the development of fourth ventricular entrapment. Preterm infants have morphologically different skull dimensions which predispose them to develop a symptomatic TFV due to their small posterior fossa. The role of long-term overdrainage as a risk factor in the development of TFV is questionable. TFV can also follow surgery of tumors in the fourth ventricle due to possible scarring of the fourth ventricular outlet foramina. Although we had only two patients in our cohort who received aqueductoplasty with stenting but according to their clinical outcome, we conclude that both fourth ventricular shunting and endoscopic aqueductoplasty with stenting are effective in managing TFV. The role of endoscopic aqueductoplasty in managing TFV is in our opinion very important and should be considered first option whenever possible. On the contrary, microsurgical fourth ventriculostomy is not recommended due to its high failure rate, possibly caused by additional scarring through the surgery. Finally, early detection and intervention may help in avoiding fatal complication and improving the neurological function. Further research is highly required to investigate the details of CSF microcirculation and its possible role in the development of PHH. Accordingly, future management of neonatal hydrocephalus may change markedly.

## Abbreviations

CSF: Cerebrospinal fluid
CT: Computed tomography
GMH: Germinal matrix hemorrhage
IVH: Intraventricular hemorrhage
MRI: Magnetic resonance imaging
PHH: Post-hemorrhagic hydrocephalus
TFV: Trapped fourth ventricle
